# Transcriptome analysis provides insight into prickle development and its link to defense and secondary metabolism in ***Solanum viarum*Dunal**

**DOI:** 10.1038/s41598-018-35304-8

**Published:** 2018-11-20

**Authors:** Shatrujeet Pandey, Ridhi Goel, Archana Bhardwaj, Mehar H. Asif, Samir V. Sawant, Pratibha Misra

**Affiliations:** 1 Council of Scientific and Industrial Research - National Botanical Research Institute, Lucknow, 226001 India; 2 Academy of Scientific and Innovative Research (AcSIR), Ghaziabad, 201002 India

**Keywords:** Prickles, Viarum Dunal, Glandular Trichomes (GTs), Differential Transcriptome Analysis, Abiotic Stress Management, Plant development, Plant molecular biology

## Abstract

Prickles are epidermal outgrowth found on the aerial surface of several terrestrial plants. Microscopic studies on prickles of *S*. *viarum* Dunal indicated a crucial role of glandular trichomes (GTs) in their development. A spontaneously obtained prickleless mutant showed normal epidermal GTs, but its downstream developmental process to prickle was perturbed. Thus, prickleless mutant offers an ideal opportunity to unveil molecular regulators working downstream to GTs in the prickle formation. Differential transcriptome analysis of epidermis of prickly and prickleless mutant revealed that expression of several defense regulators like ethylene, salicylic acid, PR-proteins, etc. were significantly down-regulated in prickleless mutant, provide an important link between defense and prickle development. It was also noteworthy that the expression of few essential development related TFs like MADS-box, R2R3-MYB, REM, DRL1, were also down-regulated in the stem, petioles, and leaves of prickleless mutant indicating their potential role in prickle development. Interestingly, the gene expression of terpenoid, steroid, flavonoid, glucosinolate, and lignin biosynthesis pathways were up-regulated in prickleless mutant. The biochemical and qRT-PCR analysis also confirmed metabolite elevation. These results indicated that the loss of prickle was compensated by elevated secondary metabolism in the prickleless mutant which played important role in the biotic and abiotic stress management.

## Introduction

Plants are primarily dependent on their dermal tissues for protection and defense against pathogens and predators. They utilize distinct strategies to defend themselves against pathogens and predators in the natural environment. Epidermis being the most intimate layer interfacing with the environment is highly prone to various stresses thus directly influences the fitness of the plant^1^. It utilizes both structural (eg. wax, cuticle, trichome etc.) and biochemical (eg. terpenoids, alkaloids, phenolics) mechanism to provide defense^2,3^. Some epidermal structures like, trichome, prickle, thorns deter various insects and herbivores. Furthermore, several secondary metabolites, like terpenoids, alkaloids, phenolics, glucosinolate, etc. which are produced by epidermal cells, mainly in glandular trichomes (GTs), also provide defense against herbivores^4–6^. These structural and biochemical mechanisms varies with the plant species.

*Solanum khasianum* C. B. Clarke var. *chatterjeeanum* Sen Gupta (Synonym *S*. *viarum* Dunal) is a medicinally important plant having higher content of steroidal alkaloids like solasodine, solamargine, solanidine, etc. provide major raw material for the semi-synthetic and commercial production of pharmaceutically important steroids^7,8^. Aerial parts of this plant are covered with sharp pointed prickles which make their handling difficult for large-scale cultivation. Prickles are the extensions of epidermis and sometimes cortical cells as well, and lack vasculature. Few studies on prickles revealed that they are the extensions or modifications of glandular trichomes^9,10^. There has been considerable progress made to understand the molecular processes behind differentiation of epidermal cells into unicellular trichomes and root hair in *Arabidopsis*^11–13^. Basically, the complexes of the members of three gene families MYB, bHLH and WD40 play crucial role in cell fate determination of unicellular trichomes^11^ but the information regarding molecular regulators associated to GTs are still in infancy^13–15^. Recently, two SSR markers closest to non-glandular multicellular trichomes (the term spine was used for it in cucumber) determining the trait for trichome density has been identified in cucumber and the potential gene for the trait was predicted as the member of WD40 TFs family^16^. Parallel to their prediction, function of *CsTTG*1, a homolog of WD40 repeats was identified in the regulation of cucumber fruit wart/spine/trichome development^17^. Even though these studies provided necessary foundation to understand the gene networks leading to the conversion of GT into prickle. But the molecular basis behind prickle formation and key regulators involved in differentiation of GTs into prickle are still unknown. A prickleless mutant of *S*. *viarum* was obtained spontaneously during *S*. *viarum* breeding program^18,19^ at CSIR-National Botanical Research Institute, Lucknow, India. Prickleless mutants had been adapted well to the natural environment and are growing in the field with the prickly *S*. *viarum*. These two prickly (wild type, WT) and prickleless (mutant) of *S*. *viarum* with great genetic similarity provided an ideal biological system to understand the molecular processes behind the prickle development. It also offers an opportunity to analyze the role of prickle in plant defense. In the present study, we have performed cellular characterization and RNAseq analysis of the prickly and prickleless mutant to explore molecular cues for prickle development. Our study also identified an interesting link between prickle development and secondary metabolism important for plant adaptation.

## Results

### Morphological characterization of prickly and prickleless *S*. *viarum*

**Figure 1:**
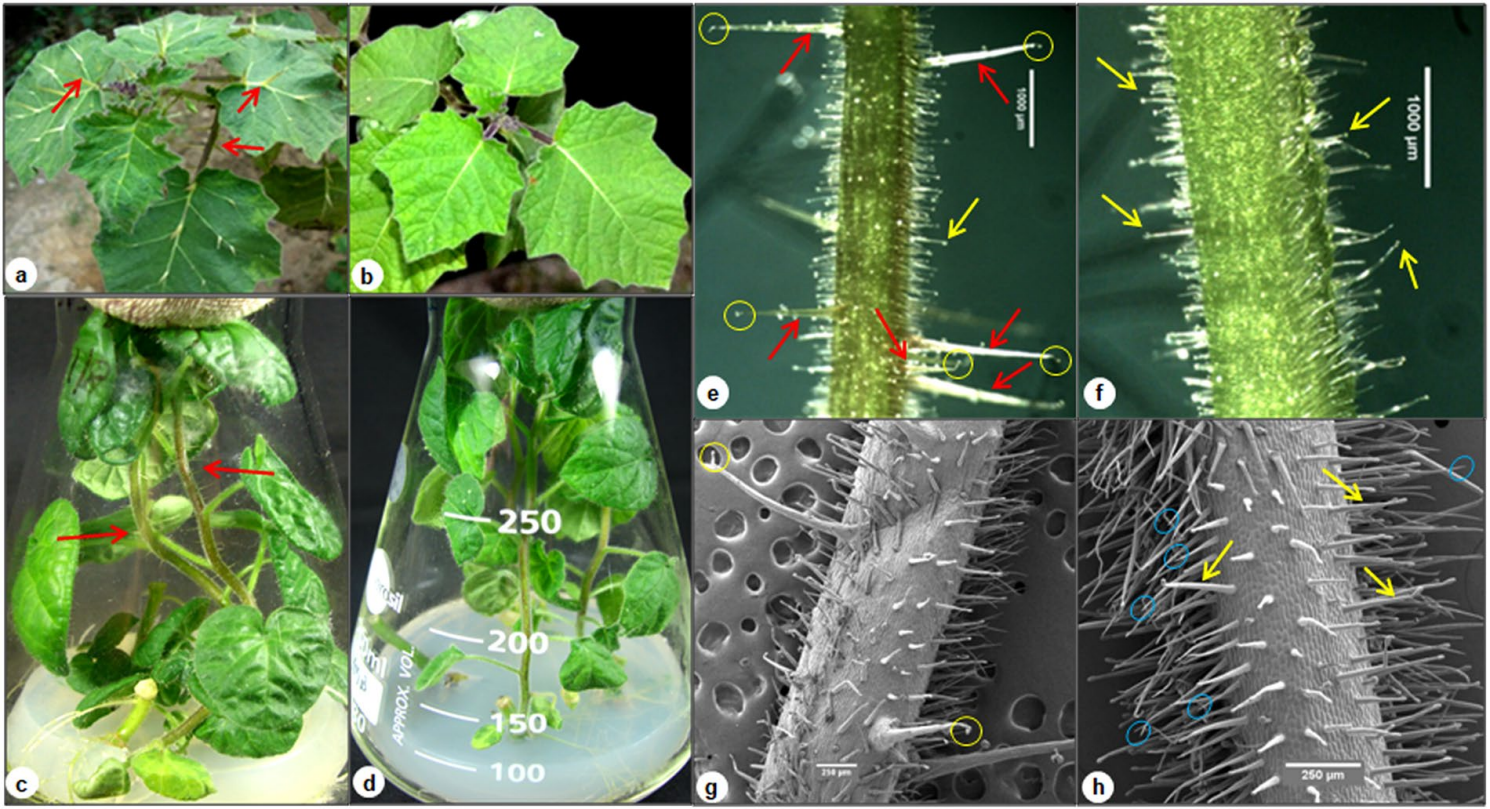
Micrographs showing prickles and trichomes on the surface of prickly and prickleless *S*. *viarum*. (**a**) Prickly and (**b**) prickleless. 45-days-old *in vitro* grown plantlets (**c**) prickly and (**d**) prickleless. Light microscopy of stem (**e**) prickly and (**f**) prickleless. SEM micrographs showing (**g**) prickles and trichomes on the prickly stem and (**h**) trichomes on the prickleless stem. Red arrows indicate prickle and yellow glandular trichome (GTs), yellow circle indicate GTs and blue non-GTs.

**Figure 2:**
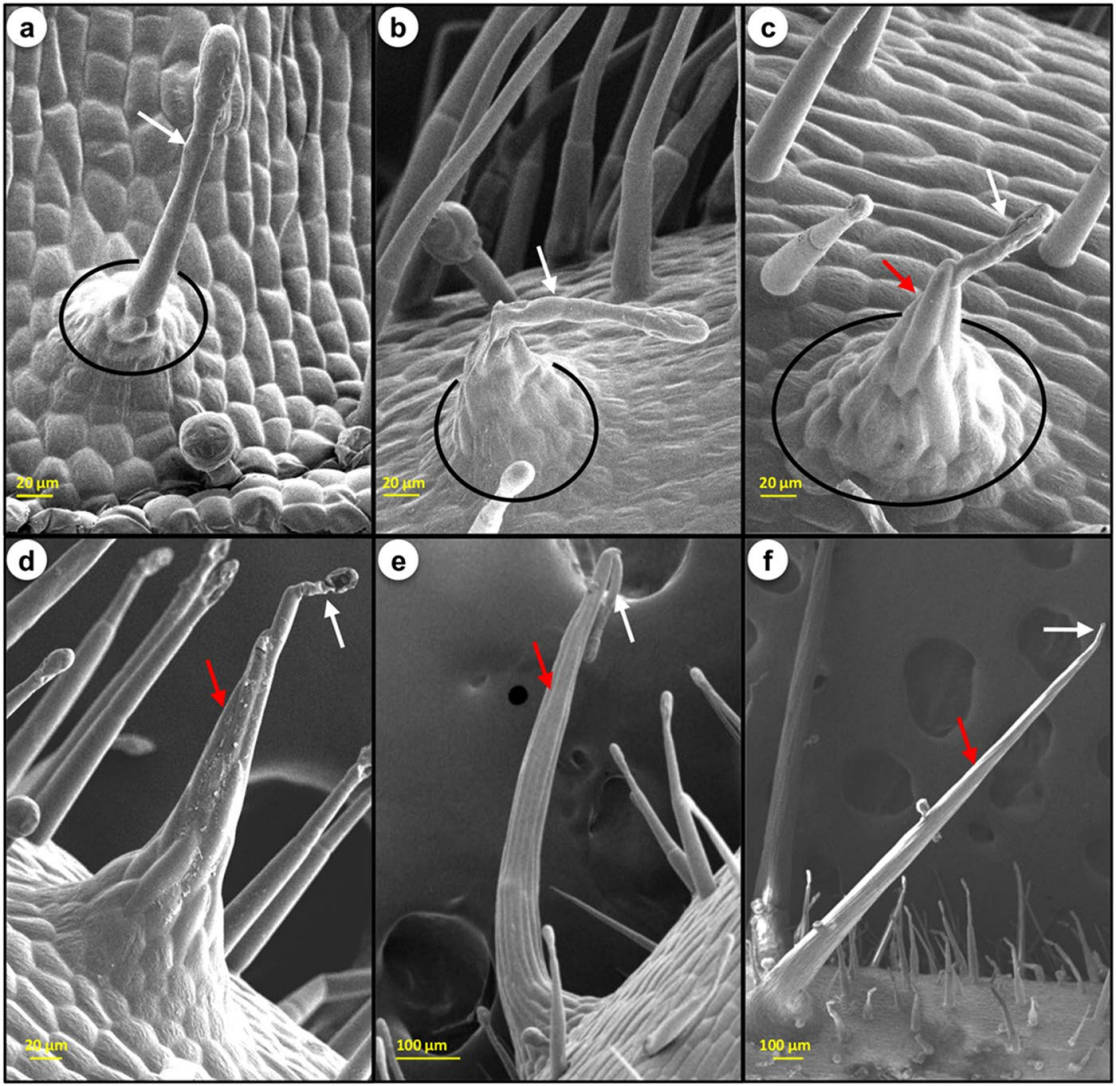
Different stages of prickle development. (**a**) Developing bulbous structure beneath the GT. (**b**) Growing basal zone beneath the GT. (**c**) Emerging prickle. (**d**–**f**) GT attached to the tip of growing prickles. Red arrows indicate prickle, white trichome and circle the basal area.

Prickly and prickleless mutant of *S*. *viarum* differ phenotypically on the basis of the presence of prickles on their aerial surface, like stem, petioles and leaves (Fig. 1a–h). Prickly stem when examined under light microscope revealed a specific character of prickles that they have a glandular trichome associated to their tip (Fig. 1e). Although, non-GTs were also present on the surface of the stem but they were not seen to be associated to the tip of any prickle. The prickles were abundant on the surface of the stem and thus used for further detailed analysis (Fig. 1c). To get insight, we have performed scanning electron microscopy of the stem which revealed that surface of prickly WT (Fig. 1g) was covered with dense multicellular GTs, non-GTs and the prickles, whereas prickleless mutant was covered with multicellular GTs and non-GTs (Fig. 1h). Structurally, prickles were multicellular, much larger, harder and sharper than trichomes (Supplementary Figs S1,S2). Similar to trichomes, prickles of *S*. *viarum* were also multicellular in structure, made up of vertically elongated cells but acquired huge and larger basal area than the trichomes (Supplementary Figs S1,S2) and every prickle has a GT associated to its tip (Supplementary Fig. S2; Fig. 2d–f). Mature prickles also have few GTs (type-IV GT with four-celled head) on their surface (Figs 1g, 2f).

### Developmental stages and cellular characterization of prickles

Prickle development in *S*. *viarum* was observed tightly associated to GTs. Prickle formation initiated only beneath the GTs (Fig. 2a; Supplementary Fig. S2) and completed into 3 major stages. First, the formation of base - initially, a bulbous structure started to develop around the base of GT (Fig. 2a) which grew in size to provide a larger basal area (Fig. 2b). Second, the emergence of prickle - the central cells of the basal area arranged vertically, grew and divide anticlinally, just below the GT (Fig. 2c; Supplementary Fig. S2). Third, the vertically elongated cells divide, grew and hardened to develop into a larger glandular prickle (Fig. 2d–f; Supplementary Fig. S2). GT remained attached to the tip of the prickle (Fig. 2, Supplementary Fig. S2). To examine the cellular origin of prickle, the transverse sections of stem were observed under light microscope which revealed that prickles of *S*. *viarum* contained epidermal and adjacent hypodermal cells (Supplementary Fig. S2). Head, stalk and basal cells of GT associated to prickle have dense cytoplasm (Supplementary Fig. S2a) which suggested their higher metabolic activity. Epidermal and hypodermal cells just beneath the GT were divided and elongated in order to differentiate into prickle forming cells (Supplementary Fig. S2a,b). Prickle forming cells were also elongated similar to stalk cells of trichome (Supplementary Fig. S2c). It was also observed that GTs remained attached to the tip of the prickle and they were not modified during prickle development. These results suggested that prickles of *S*. *viarum* are not the modification of GTs instead these GTs might be providing some signals to induce the prickle and thus became very crucial for their formation.

### Sequencing and characterization of transcriptome of *S*. *viarum*

To understand the molecular cues responsible for the prickle development, we have performed RNA-seq of the epidermal layer of prickly and prickleless mutant. A total of 1,56,926 transcripts were predicted by illumina paired end sequencing and analysis. The total of 43.17% of these transcripts was annotated with the *S*. *lycopersicon* and 40.13% with *Arabidopsis* protein databases (Supplementary Table S1). Functional classification of these transcripts through GO enrichment analysis revealed that the transcriptome was mainly dominated by the metabolic activity and the interaction of the epidermal tissues with their intimate environment (Supplementary Fig. S3). Gene related to cellular processes, metabolic processes, responses to stimulus, biological regulation, regulation of biological processes were the key biological processes enriched in the epidermal tissues. Catalytic and binding terms of molecular function, and cell, cell part and organelles terms of the cellular component were also enriched (Supplementary Fig. S3).

### SNP analysis for the genetic relatedness of prickly and prickleless mutant

To identify the genetic relatedness between prickly and prickleless mutant of *S*. *viarum*, we have performed the SNPs analysis. Prickly and prickleless mutant were found 99.92% genetically similar. At the stringency level described in material & methods, we have found 150 bi-allelic SNPs in 95 contigs which represent only 0.086% of the total contigs. Twenty-six contigs have two or more SNPs (Supplementary Table S2). The SNPs analysis thus revealed that prickly and prickleless mutant used in the study are genetically related with probably minor difference which may reflect their prickliness. In further analysis, of these 95 contigs, 23 were found to be differentially expressed between prickly and prickleless mutant. Among these SNPs containing differentially expressed genes, a development related gene DRL1 was among the top 15 down-regulated genes in epidermis of prickleless mutant (Supplementary Table S4).

### Differentially expressed genes (DEGs)

**Figure 3:**
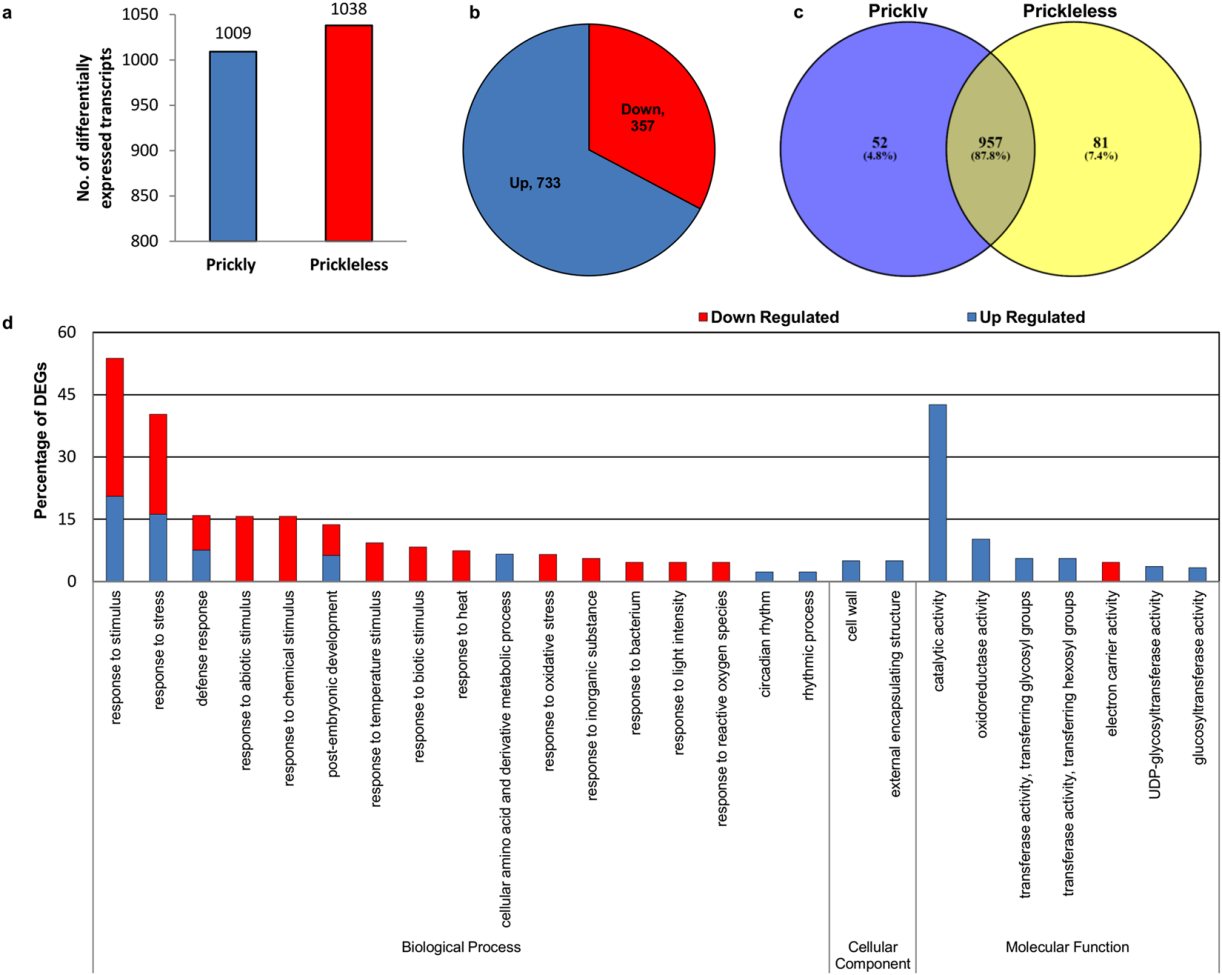
Analysis of the DEGs between prickly and prickleless epidermis. (**a**) Number of DEGs. (**b**) Number of Up and down-regulated genes. (**c**) Distribution of commonly and uniquely expressed genes. (**d**) Enrichment of DEGs in top 15 up and 15 down-regulated GO categories in the prickleless epidermis. Lowest *p*-value was considered to select top GO categories.

A total of 1,937 statistically significant unigenes were found differentially expressed in epidermal tissues of prickleless in comparison to prickly (Supplementary Table S3). Of the 1,937 unigenes, only 1,090 with the fold change < −1.5 and >1.5 were used for further functional evaluation. Among the 1,090 unigenes; 733 up-regulated and 357 down-regulated unigenes were found in the epidermis of prickleless mutant (Fig. 3b). TFs DRL1 (DEFORMED ROOTS AND LEAVES1) which regulates various plant developmental pathways were among the top down-regulated genes in the prickleless epidermis (Supplementary Table S4). Several pathogenesis-related (PR) proteins such as SN1b, Sn-2 major latex-like protein (MLP), were also among the top down-regulated genes in the prickleless epidermis (Supplementary Table S4). In addition to these, fatty acid desaturase and homocysteine S-methyltransferase 3, sgt1 (UDP-galactosyltransferase) were the top up-regulated genes in the prickleless epidermis. Most of the other highly DEGs are still uncharacterized (Supplementary Table S4).

Furthermore, 957 (87.8%) of DEGs were commonly expressed in both the prickly WT and prickleless mutant, while 52 (4.8%) genes were found completely knock down and 81 (7.8%) genes were uniquely up-regulated in the epidermal tissues of prickleless mutant (Fig. 3c, Supplementary Table S5). Putative disease resistance protein CC-NBS-LRR, 1-aminocyclopropane-1-carboxylate synthase involved in ethylene (ET) biosynthesis and some uncharacterized genes were completely knocked down in prickleless mutant whereas UDP-glucose: glucosyltransferase involved in secondary metabolite production, STH-2 (pathogenesis-related protein) defend to the pathogen and some uncharacterized genes were uniquely up-regulated in prickleless mutant (Supplementary Table S5).

### Validation of transcriptome data by qRT-PCR analysis

To validate the transcriptome analysis data generated through sequencing, 15 genes (highly up-regulated, highly down-regulated and similarly expressed) were randomly selected for the qRT-PCR analysis. Expression pattern obtained through qRT-PCR analysis and the transcriptome data analysis showed a very good correlation which reflects the accuracy of our data and expression analysis (Supplementary Fig. S4).

### Biological processes altered in prickleless mutant

To explore the biological functions of DEGs, the functional enrichment analysis was performed (Supplementary Table S7). The analysis showed that some GO terms, such as responses to stimulus (both biotic and abiotic), response to stress and response to defense were the top significantly altered “biological processes” in prickleless mutant (Fig. 3d; Supplementary Table S7). The higher percentages of DEGs were enriched to these top significant biological processes. Additionally, the percentage of down-regulated genes in these top biological processes was also higher in prickleless epidermis (Supplementary Fig. S4d). Most of the top differentially expressed biological processes were related to plant defense and stress, therefore, they were considered the most relevant biological processes associated with prickle development. The post-embryonic developmental process was also among the top altered biological processes (Fig. 3d; Supplementary Table S7). Further, some genes related to response to stimulus, biological regulation, etc. were uniquely expressed in the prickly WT (completely knock down in prickleless mutant), whereas various transcripts related to distinct GO terms, such as cellular metabolic process, etc. were found to be uniquely expressed in prickleless mutant (Supplementary Table S8).

### Metabolic pathways altered in prickleless mutant

Several metabolic pathways were significantly altered in prickleless mutant (Supplementary Table S9). Few pathways, such as flavonoid, steroids, glucosinolate were among the top 15 significantly up-regulated pathways in the prickleless epidermis (Supplementary Fig. S5a). A total of 26 up-regulated genes were enriched to the secondary metabolite biosynthesis pathways (Supplementary Fig. S5a). Ethylene biosynthesis, gibberellins inactivation via 2-β hydroxylation, diterpenoid biosynthesis and methionine metabolism were the top significantly down-regulated pathways (Supplementary Fig. S5a). Additionally, KOBAS analysis of the uniquely expressed unigenes revealed that some genes related to glucosinolate biosynthesis were uniquely expressed in prickleless mutant and some genes related to ethylene biosynthesis pathways were uniquely expressed in prickly WT (Supplementary Fig. S5b).

**Figure 4:**
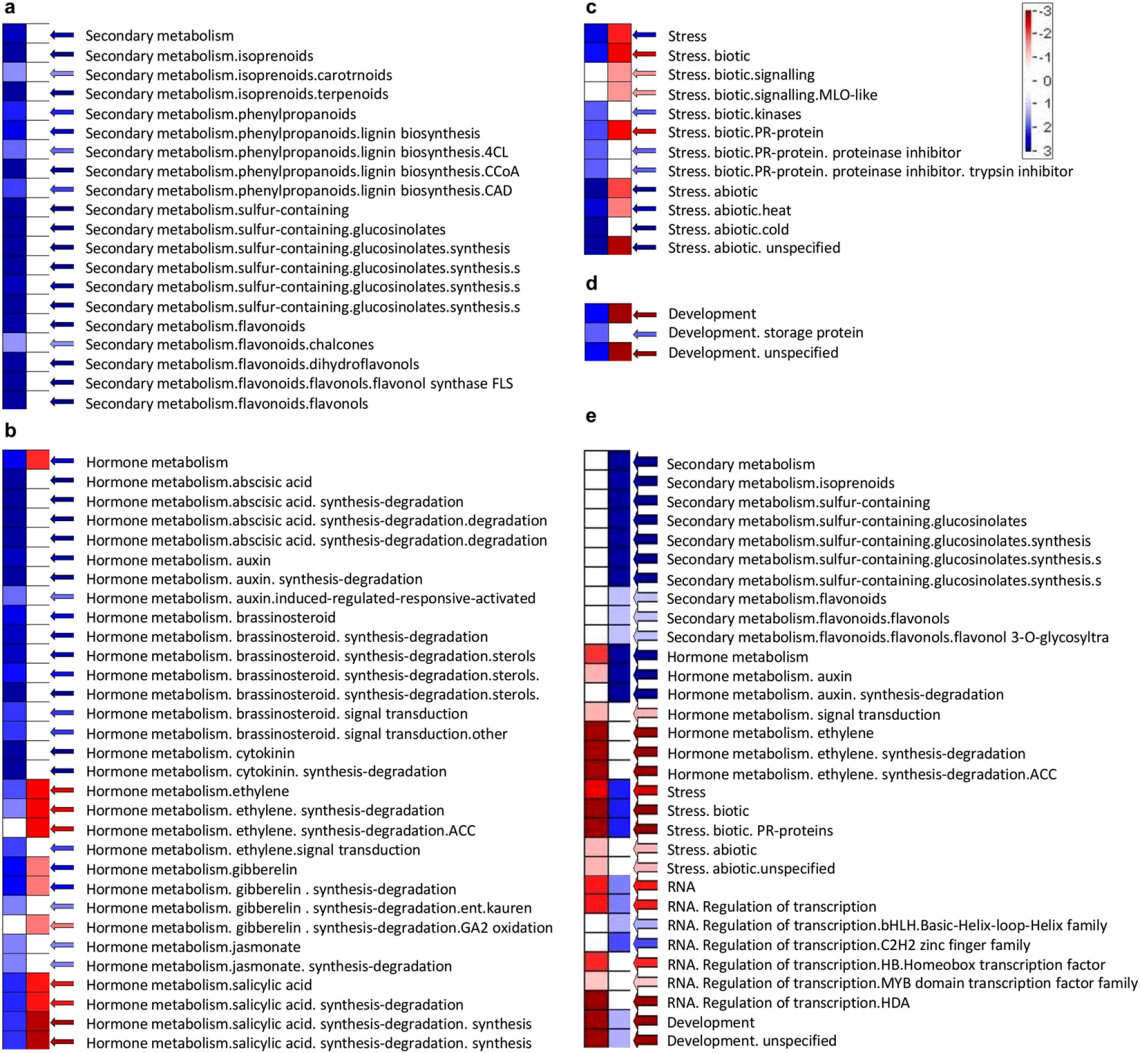
Enrichment analysis of the differentially and uniquely expressed genes using PageMan tool to represent the detailed expression pattern of genes associated to different processes. (**a**–**d**) DEGs, (**a**) Secondary metabolism, (**b**) Hormone metabolism, (**c**) Stress associated, and (**d**) Development related genes. (**e**) Uniquely expressed genes.

Furthermore, to visualize the detailed expression pattern of the transcriptome in various metabolic processes, PageMan analysis of DEGs was performed (Supplementary Fig. S6). Several pathways were found up or down-regulated in prickleless in comparison to prickly *S*. *viarum* (Supplementary Fig. S6). Similar to previous results differences in expression patterns of the development, stress, hormone metabolism and secondary metabolism associated genes were more considerable (Fig. 4). Transcripts associated to various secondary metabolite biosynthesis (Fig. 4a) and abiotic stresses (Fig. 4c) were highly up-regulated whereas transcripts associated to biotic stresses (Fig. 4c) and some unspecified developmental pathways (Fig. 4d) were highly down-regulated in prickleless epidermis. Several genes associated to various hormone metabolisms like auxin, cytokinin, brassinosteroid, abscisic acid and jasmonate were observed up-regulated whereas ethylene and salicylic acid (SA) metabolism associated (related to biotic stress) genes were down-regulated in prickleless mutant (Fig. 4b).

PageMan analysis of uniquely expressed unigenes of prickly and prickleless mutant was also performed (Supplementary Fig. S7). Some genes associated with secondary metabolism were expressed uniquely in prickleless epidermis (Fig. 4e). Some unspecified developmental pathways related genes were highly expressed uniquely in prickly WT (Fig. 4e). One member of the homeobox TFs families was found to be uniquely expressed in prickly WT. Some genes related to hormone metabolism, such as ethylene metabolism were highly expressed only in prickly WT, whereas genes related to auxin metabolism were uniquely expressed in prickleless mutant (Fig. 4e). Some biotic stress associated genes were also found to be highly expressed only in prickly WT (Fig. 4e).

**Figure 5:**
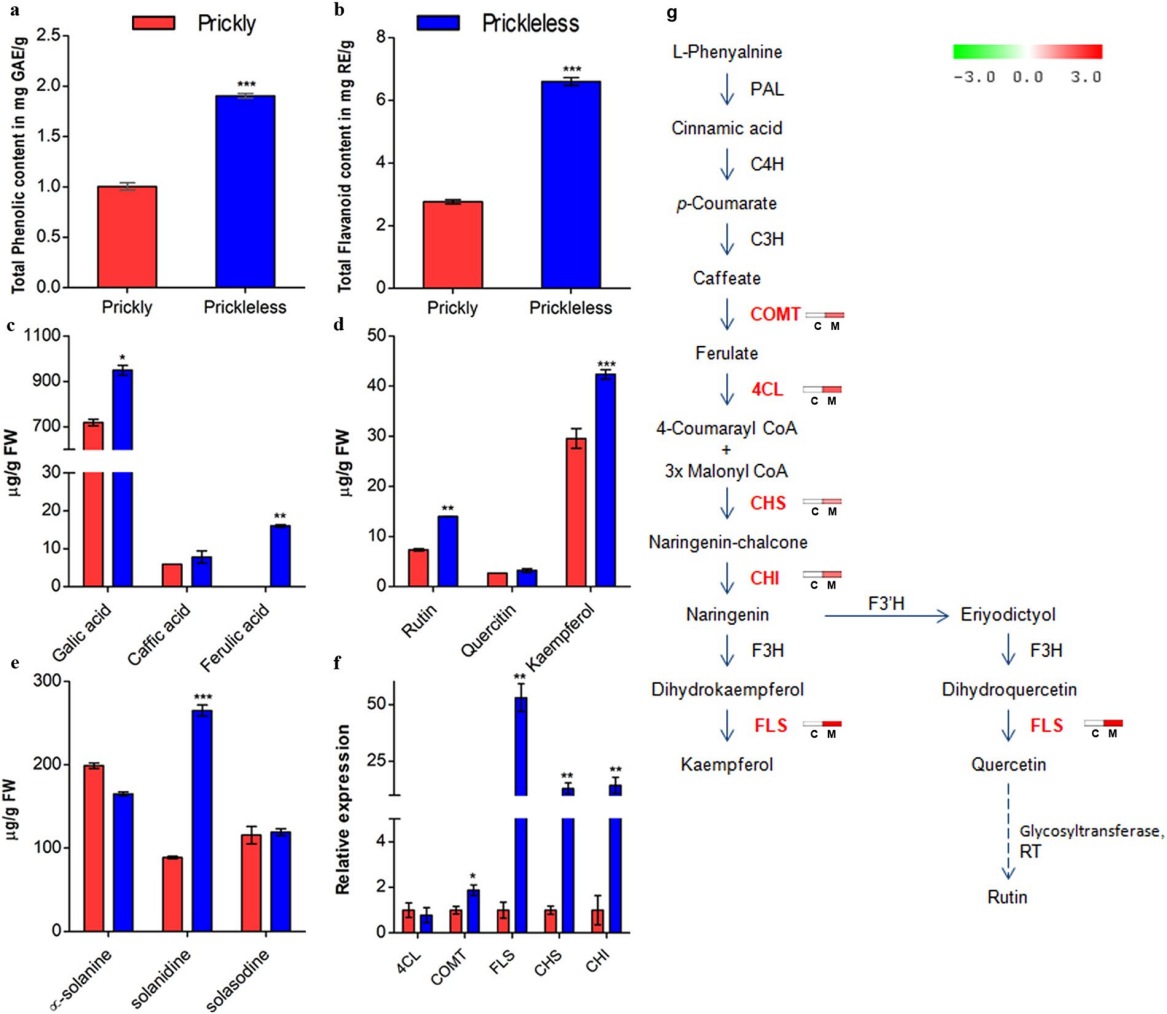
Quantification of secondary metabolite level and expression analysis of related genes in the epidermis. (**a**,**b**) Spectrophotometric quantification. (**c–e**) Quantification through HPLC. (**f**) qRT-PCR analysis of phenolics and flavanoid related genes. (**g**) Schematic representation of phenolics and flavonoid biosynthesis pathways showing the heat map of specific genes altered in mutant (WT indicated by C and prickleless by M). PAL, L-phenylalanine ammonia-lyase; C4H, cinnamate 4-hydroxylase; C3H, coumarate 3-hydroxylase; COMT, caffeic acid *O*-methyltransferase; 4CL, coumarate CoA ligase; CHS, chalcone synthase; CHI, chalcone isomerase; F3H, flavanone 3-hydroxylase; F3′H, flavonoid 3′-hydroxylase; FLS, flavonol synthase; RT, rhamnosyltransferase. Dashed arrows represent multiple enzymatic steps. Error bars represent the SD of triplicates and *represents statistical significance analyzed by t-test.

Since our prickleless mutant showed typical up-regulation of genes belonging to several secondary metabolite biosynthesis pathways, we decided to validate this by biochemical characterization of both prickly and prickleless mutants. Thus, we quantified the total phenolics and flavonoids in epidermal tissues spectrophotometrically and found the significantly higher level of both phenolics and flavonoids in prickleless mutant (Fig. 5a). Furthermore, the concentration of several phenolics and flavonoids derivatives estimated through HPLC revealed that the concentration of gallic acid, ferulic acid, rutin, and kaempferol were significantly higher in the epidermis of prickleless mutant (Fig. 5b, Supplementary Fig. S8). We have also estimated the concentration of medicinally important alkaloids (Fig. 5c, Supplementary Fig. S8) found in the berries of *S*. *viarum*, although, transcriptome analysis did not indicate any differences about their biosynthetic pathways due to lack of information. These alkaloids were analyzed first time in the epidermis (might be in GTs) of *S*. *viarum*. The concentration of solanidine was estimated higher in the prickleless epidermis in comparison to prickly WT (Fig. 5c). The qRT-PCR analysis of caffeic acid *O*-methyltransferase (COMT) and 4-coumarate:CoA ligase (4CL) genes related to phenolic biosynthesis and flavonol synthase 1 (FLS), chalcone synthase (CHS) and chalcone flavanone isomerase (CHI) genes related to flavonoid biosynthesis pathways was performed. Biosynthetic pathway of these key enzymes along with the heat map showing their expression pattern was shown in Fig. 5g. The results revealed that they were highly expressed in prickleless epidermis (Fig. 5g). However, the qRT-PCR analysis of alkaloid biosynthesis could not be performed due to unavailability of marker genes associated to solanidine biosynthesis.

### Putative transcription factors expressed differentially in prickleless mutant

**Figure 6:**
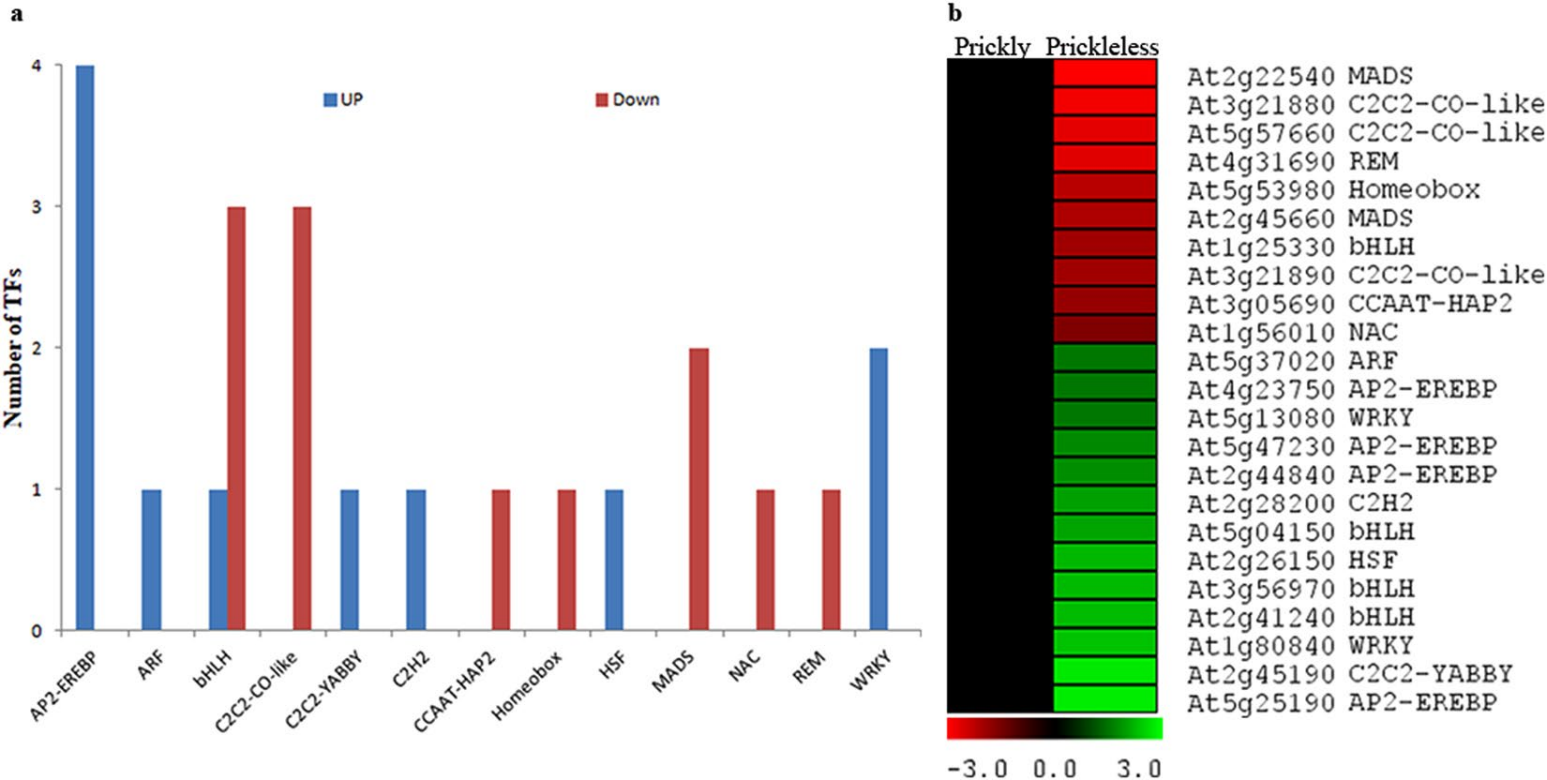
Analysis of differentially expressed TFs in the prickleless mutant in comparison to prickly. (**a**) Number of the members of different TFs families. (**b**) Expression patterns of the members.

To identify the differentially expressed TFs, we have compared the sequences IDs of DEGs with AtTFDB. Twenty four members of 13 different TFs families were found to differentially expressed in the prickleless epidermis (Fig. 6). The member of MADS-box, homeobox, REM, NAC, CCAAT-HAP2, C2C2-CO-like, bHLH families TFs were found down-regulated in the prickleless epidermis (Fig. 6). The members of these TFs families are well known to play a key role in plant growth and development. Several members of AP2-EREBP, bHLH, WRKY, HSF, ARF, etc. were up-regulated in prickleless mutant (Fig. 6). Members of these families also regulate plant growth and development and help to cope with the environmental stresses.

### Potential candidate genes associated to prickle development

**Figure 7:**
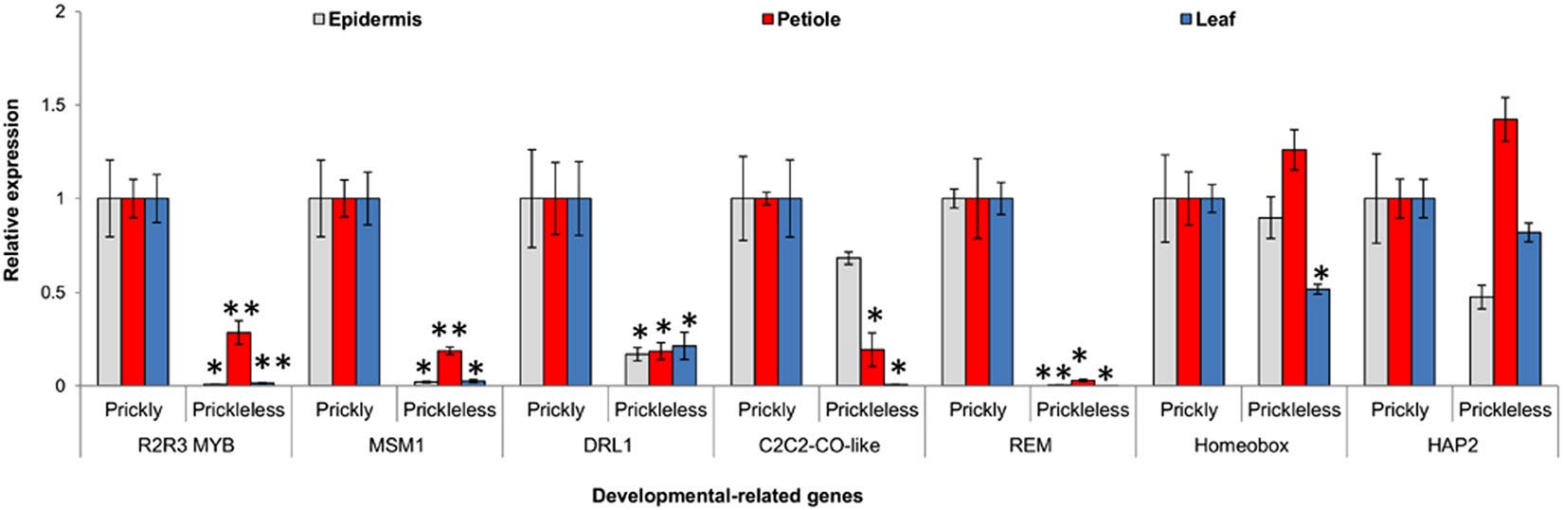
Relative expression patterns of the plant development-related genes in various tissues of prickly and prickleless mutant. Error bars represent SE of means of triplicates.

We hypothesized that loss of prickly phenotype could be due to the knockdown or knockout of some prickle development specific genes in prickleless mutant. We have found some TFs regulating distinct developmental pathways were down-regulated in prickleless mutant. Two of them, DRL1 (not detected by *At*TFDB) and MSM1 (MADS-box TFs), playing various important roles in plant development, were among the list of the top down-regulated genes (Supplementary Table S4). Expression profiles of these down-regulated TFs were analyzed in the entire prickle bearing tissues of *in vitro* grown plantlets via qRT-PCR analysis. Members of some transcriptional regulators family, such as REM (B3 TF family member), R2R3-MYB TF (not detected by AtTFDB), MSM1 (MADS-box TF) and DRL1 TFs were significantly down-regulated in all the prickle bearing tissues of prickleless mutant (Fig. 7). These TFs could be the potential key regulators of the prickle development.

## Discussion

Glandular trichomes are multicellular outgrowth of epidermal tissues having a secretary cell at their tips known as the head^14^. The transcriptional network regulating the development and patterning of unicellular trichomes is well characterized in *Arabidopsis*, however the understanding of development of glandular trichomes is still lacking in plants^13–15^. Studies on prickle development has suggested that prickles either develop directly from glandular trichome (e.g., raspberry) or the signal coming from GTs induces the prickle development (e.g., blackberry)^9^. Recently, morphological characterization of prickles of *Vitis davidii* has also shown prickles as modified glandular trichome^10^. The microscopic studies on *S*. *viarum* suggested that GTs might be playing some important role in prickle development because prickle formation initiated only beneath the GTs. It is noteworthy here that prickleless mutant also has the similar GTs but unable to induce prickles. It may, therefore, be presumed that there are some additional factors governing the prickle differentiation working downstream to GTs or similar to blackberry, some signal coming from GTs might be responsible to induce prickles. Prickles were epidermal in origin and hypodermal cells were also found to be involved in their formation, similar to as reported in blackberry^9^ and *Vitis davidii*^10^.

In *S*. *viarum*, visibility of prickles started on the stem’s surface after 10–15 days of seed germination or at 4-5 leaf stage of the seedlings. However, trichomes are present from the very beginning and could be observed at the surface of even the first cotyledon. The development of prickles is a non-synchronous process and different stages of prickles could be seen interspersed and their formation continued with the plant growth.

**Figure 8:**
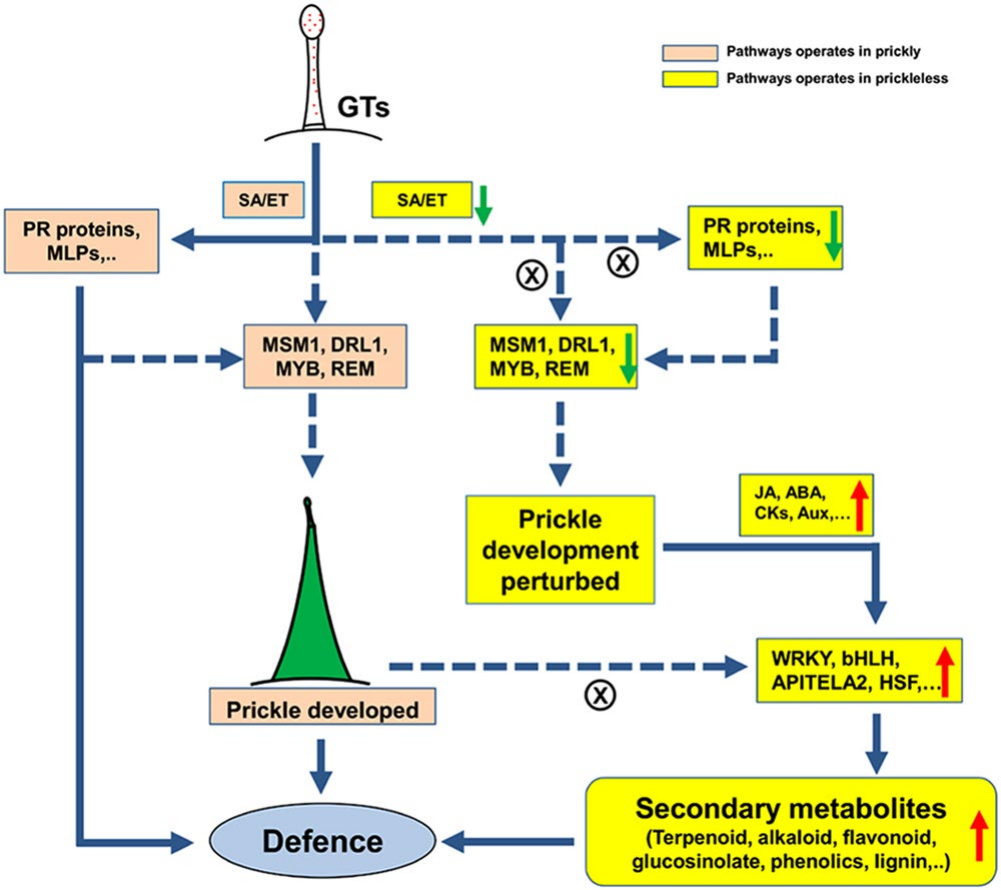
Putative layouts showing the summary of the possible signaling pathway governing prickle development from GT and enhance the secondary metabolism. Green arrow indicates down-regulation, red arrows up-regulation and dashes arrow putative pathways.

Trichomes and prickles both are evolved independently in several plants to provide protection against herbivores^20^ and also abiotic stresses^21^. Therefore, it was expected that loss of prickly phenotype should greatly affect the stress associated responses of prickleless mutants. Differential transcriptome analysis of epidermal layers of prickly and prickleless mutants revealed that two defense related hormones, ethylene and salicylic acid biosynthesis related genes were highly expressed in prickly epidermis than prickleless under normal conditions (Fig. 4b). Ethylene and salicylic acid are synthesized in the infected or wounded tissues to provide defense^22–24^ but some reports have also suggested their essential role in plant development^25^. SA and ET induced several PR proteins have been identified^26,27^. We have also found several pathogenesis related PR 10 type protein and their homolog major latex-like protein (MLP151)^24^, cysteine-rich PR proteins: non-specific lipid transfer protein (nsLTP)^28^, disease resistance protein CC-NBS-LRR^29^, Sn-2 protein (MLP like protein), etc. highly up-regulated in the prickly epidermis (Supplementary Table S3; Table S4). These pathogenesis related proteins are usually expressed against infection or wounding in plants but they are also reported to be expressed in the trichomes of tobacco under normal conditions^30^. These results suggested the positive role of defense related hormones, SA and ethylene, and these hormone mediated PR proteins in prickle development (Fig. 8). As we have found that GTs have some important role in prickle development, the higher expression of these defense related genes (located in GTs) in prickly epidermis indicated the potential role of these biotic stress related genes upstream to prickle development in *S*. *viarum* (Fig. 8). It might be possible that they provide the micro environment to epidermal tissues which enforces the development of prickle to protect them against grazing animals.

Several genes associated with secondary metabolite biosynthesis were analyzed significantly up-regulated in prickleless mutant (Fig. 4a). Flavonoids, phenolics and alkaloids concentrations were estimated higher in epidermal tissues of prickleless mutant (Fig. 5). Few abiotic stress associated transcription factors like AP2/EREBP^31^, bHLH^32^, WRKY^33–35^, HSF (heat shock TFs), etc. were found to be overrepresented in prickleless mutant. Several reports have suggested role of these TFs in the biosynthesis of various secondary metabolites like terpenoides^36^, alkaloids^37,38^, etc. in the plants. It could be suggested from these results that a putative pathway is being operated in prickleless epidermis where the stress related TFs are involved in induction of secondary metabolite related biosynthetic genes to enhance their production (Fig. 8). These secondary metabolites may help in protection of prickleless plants against various biotic and abiotic stresses because their role in plant defense is well known^6^. Glandular trichomes are reported to synthesize and store plenty of secondary metabolites, such as terpenes, phenylpropanoid derivatives, flavonoids, alkaloids, etc. to provide defense against biotic stresses^4,5,39^. Therefore, it could be inferred from these results that prickleless mutant compensates the unavailability of one defense mechanism (prickles) by enhancing the other mechanism (secondary metabolite) to sustain in the environment. Epidermal cells of prickleless mutant were diverted towards the production of secondary metabolites to defend the plant as do prickles in prickly WT. These results indicated a negative correlation between the prickle development and secondary metabolite biosynthesis in epidermal tissues.

The molecular analysis of unicellular trichomes of *Arabidopsis* have revealed that the members of three gene families, MYB, bHLH and WD40, play crucial role in trichome development^11,40^. Several genes from these gene families, such as TRANSPARENT TESTA GLABRA1 (TTG1), GLABRA3 (GL3), ENHANCER OF GLABRA3 (EGL3), WEREWOLF (WER) involved in regulating trichome development have been characterized in *Arabidopsis*^41^. Recently, role of *CsTTG*1, a homolog of WD-40 repeats, in regulation of the density of spine or multicellular trichomes was characterized in cucumber^17^. Several genes associated to plant development have been identified which were differentially expressed in epidermal tissues of prickleless mutant in comparison to prickly *S*. *viarum*. We presume that loss of prickly phenotype may be due to partial or complete loss of prickle development associated genes in prickleless mutant. Transcriptome analyses have shown that members of several TFs families involved in plant growth and development such as homeobox, MADS-box^42^, REM^43^, MYB^13,40^, NAC^44^, HAP2 transcription factors^45–47^, C2C2-CO-like^48^ were down regulated in prickleless mutant (Fig. 6). Furthermore, MADS-box, R2R3-MYB, DRL1 and REM TFs were significantly down regulated in all the prickle bearing tissues of prickleless mutant (Fig. 7). Therefore, MADS-box, R2R3-MYB, DRL3, REM TFs could be the potential key regulators of prickle development.

Based on our analysis we have proposed a putative pathway governing the prickle development in *S*. *viarum* (Fig. 8). According to that, GTs might be involved in induction of prickle development in *S*. *viarum*. The defense regulators like ET, SA, PR-proteins, MLP, etc. expressed highly in prickly epidermis may positively regulate prickle developmental pathway (Fig. 8). The MSM1 (MADS-box TFs), R2R3-MYB, DRL1 and REM TFs are the potential key regulators and might be working downstream to GTs. The simultaneous loss of prickle development and expression of defense related gene in the prickleless mutant is compensated by elevated secondary metabolism which play important role in the biotic and abiotic stress management. Prickleless mutants produced comparatively higher concentration of several secondary metabolites, such as phenolics, flavonoids, alkaloids, etc. in the epidermal tissues to defend the plant against herbivores thus establishing a correlation between prickle development and secondary metabolite production. Our study thus provides a novel insight into prickle development which will help to determine the molecular genetic network underlying the control of trichome development in *S*. *viarum* and other related plants.

## Material and Methods

### Plant material

Prickly wild type (WT) and prickleless mutant of *S*. *viarum* Dunal (Synonym of *S*. *khasianum* C. B. Clarke var. *Chatterjeeanum* Sen Gupta) were used as plant material in the present study. Prickleless mutant was obtained spontaneously under *S*. *viarum* breeding program at CSIR-National Botanical Research Institute, Lucknow, India and named as NBRI-Sel^18,19^ which latterly christened as ‘Nishkantak’. Shoot cultures of prickly genotype and prickleless mutant are being maintained under *in vitro* condition for last ~35 years at the host laboratory. They are also grown in natural environment under field conditions.

### Light and scanning electron microscopy

Stems of both the prickly and prickleless mutant were cut into appropriate size (~1 cm), washed twice in 0.1 M sodium cacodylate buffer and fixed in 2.5% glutaraldehyde and 4% paraformaldehyde overnight at 4 °C. Samples were washed in 0.1 M sodium cacodylate thrice, each for 20 min. Dehydration of samples was done in acetone series 15%, 30%, 60%, and 90% and 3 changes were given in 100% for 20 min each. Further, samples were dehydrated till they reach the critical point (CPD), coated with gold particles (2 coating) and observed under scanning electron microscope (FEG450 Quanta, Netherland).

Transverse sections of stem of prickly WT were cut to perform the histological studies of prickle development. Sections were stained with 0.1% aqueous safranine. To remove the excess stain, sections were washed in water several times. Semi-permanent slides were mounted in glycerine and observed under light microscope (EVOS FLc, life technology).

### RNA isolation and quality control

The epidermis of both the prickly and prickleless mutant from 45 days old *in vitro* grown plantlets was pealed out separately in liquid nitrogen. Epidermis from five plants were pooled to isolate the RNA for one biological replicate. Two such independent replicates for both prickled and prickleless mutant were used for RNA extraction and RNAseq. Total RNA was isolated using Spectrum Plant Total RNA Kit (Sigma-Aldrich, USA) and treated with the RNase free DNaseI (Ambion, Invitrogen) to remove DNA contamination. The quality and quantity of total RNA were analyzed by agarose gel, spectrophotometric analysis (ND1000, NanoDrop Technologies, USA) and Agilent 2100 Bioanalyzer RNA chip (Agilent Technologies Inc., Santa Clara, CA).

### Construction of cDNA library and Illumina deep-sequencing

The cDNA libraries were generated using mRNA assay for sequencing on Illumina HiSeq 2500 sequencing platform. Both the prickly and prickleless mutant of *S*. *viarum* were used in two biological replicates for RNA-seq experiments.

### De novo assembly, sequence clustering, annotation and transcriptome characterization

All the paired-end read sequences for both the prickly and prickleless mutant were generated from Illumina sequencing HiSeq 2500. The reads sequences were trimmed to remove the adapter sequence and NGS QC Tool Kit was used for the filtration of high-quality reads with 101 bp length. These High-quality reads were de-novo assembled using Trinity assembler with the minimum transcript length of 201. All the assembled transcripts were annotated with the protein sequences of NCBI-NR database, *Solanum lycopersicon* (www.solgenomics.com) and *Arabidopsis thaliana* genome (TAIR10, https://www.arabidopsis.org/) using blast with e-value <10^–5^. To retrieve the detailed GO annotation, all the genes expressed in the epidermis of *S*. *viarum* were analyzed through agriGo (http://bioinfo.cau.edu.cn/agriGO/analysis.php) using Singular Enrichment Statistical analysis.

### Differential gene expression and functional analysis

To perform differential expression analysis assembled transcripts of both prickly (109934 transcripts) and prickleless (103851 transcripts) mutant were clustered using CD-HIT with 99% identity threshold, which generated total 156926 transcripts. All the high-quality reads were mapped on the generated transcripts and were statistically analyzed with Cufflinks in combination with Cuffmerge and cuffdiff. All the genes with P-value < 0.05 and FDR <0.05 with fold change <−1.5 and >1.5 were selected.

To retrieve the detailed GO annotation, all the DEGs with fold change (<−1.5 and >1.5) were analyzed with agriGO (http://bioinfo.cau.edu.cn/agriGO/analysis.php) using Singular Enrichment Statistical analysis. Additionally, GO analysis of all the uniquely expressed genes in both the prickly and prickleless mutant was also performed.

### Pathway analysis and identification of transcription factors

To identify the significant pathways, all the TAIR10 ids were analyzed with KOBAS 2.0 server (http://kobas.cbi.pku.edu.cn/home.do) to retrieve the significant pathways for both up and down regulated genes and in the same way for the unigenes expressed either in prickly or prickleless mutant. All the pathways with p-value < 0.05 were considered as significant. The detailed pathway analysis was performed with PageMan using the DEGs (fold change < −1.5 and >1.5) which were annotated with the TAIR10 protein database. Similarly, PageMan analysis was also performed for the uniquely expressed genes. The analysis was performed with Benjamini Hochberg multiple testing correction using Average statistics method.

The significant TFs involved in each up and down-regulated set of the prickly and prickleless mutant were fetched using the mapped Arabidopsis ids from Arabidopsis Transcription factor database (http://arabidopsis.med.ohio-state.edu/AtTFDB/).

### Expression analysis through qRT-PCR

Real-time PCR was performed in 20 μl for a set of selected genes using Fast SYBR Green PCR Master Mix (ABI, USA). The list of selected genes and oligonucleotide primers (Eurofins, India) used for each gene has been listed in Supplementary Table S6. Ubiquitin gene (XLOC_050218) of *S*. *viarum* was used as the internal control for normalizing the expression. The relative expression was calculated by 2^−delta delta Ct method^.

### Single nucleotide polymorphism (SNPs) analysis

To identify putative single-nucleotide polymorphisms (SNPs) in transcript assembly, separately mapped all the high-quality short reads from each library using Tophat. Next, Samtools were used to detect the variable positions of SNPs from the consensus assembled data. We considered only those SNPs which possess the minimal mapping quality (−Q) and coverage (−d) of 20 bp.

### Extract preparation

Fifteen plantlets of prickly and prickleless mutant (*in vitro*-grown, 45 days old) were used for the extract preparation. Epidermal layers from the stems were peeled in 10 ml of HPLC-grade methanol, sonicated for 20 min, kept at room temperature overnight and this process was repeated thrice. After that methanol was evaporated and the extract was dissolved in 1 ml HPLC grade methanol.

### Quantification of total phenolics and flavonoid

Total phenolics content was determined using Folin-Ciocalteu reagent^49^. Firstly, 200 μl crude extract (1 mg/mL), 2800 μl milliQ (MQ) water and 0.5 mL of Folin–Ciocalteu reagent were added, mixed and incubated for 3 min. Then 2 ml NaCO_3_ (20% w/v) was added, incubated for 1 h in dark, and measured absorbance at 765 nm. Total flavonoid contents were measured by aluminum chloride colorimetric assay^50^. The 50 μl extracts, 1 ml methanol, 4 ml MQ and 0.3 ml of NaNO_2_ (5% w/v) were mixed and incubated for 5 min, followed by addition of 0.3 ml AlCl_3_ (10% w/v) and incubated for 6 min. After that 2 ml of 1 M NaOH was added, final volume was made up to 10 ml with MQ water and allowed to stand for 15 min, followed by measuring the absorbance at 510 nm. Gallic acid was used as standard for phenolics and rutin for flavanoids. Concentration was expressed in mg Rutin equivalents (RE) and mg Galic acid equivalents (GAE) per gram fresh weight (Fw).

### HPLC analysis

Qualitative and quantitative analyses for separation of all the compounds was achieved by HPLC Prominence of Shimadzu (Japan) comprising PDA SPD M 20 A system LC-20AD dual pump system, and SIL-20 AC Autoinjector (with cooler) furnished with a 20μLsample loop. Compounds were separated on a 250 mm × 4.6 mm, i.d. and 5 μm pore size Shimadzu RP-C18 column protected by guard column containing the same packing. For phenolics and flavonoid gradient mobile phage of 1% acetic acid (component A) and acetonitrile (component B) was used. The elution of mobile phase was linear gradient prepared from 20–35% of component B for 0–14 min, and 35–50% of component B for 14–40 min at flow rate of 0.6 ml/min, at room temperature. Polyphenols were detected at 254 nm^51^. For steroidal alkaloids, the elution of mobile phase was linear gradient prepared from triethyl ammonium phosphate buffer (pH-3.0) (component A) and acetonitrile (component B) in the ratio of 20, 25, 35, 45, 65, 20, 20 components B for 0.01, 12, 15, 17, 25, 30, 40 min at the flow rate of 2.0 ml/min. Alkaloids were detected at 205 nm and data were integrated with Shimadzu Lab solution software and results were obtained by comparison with standards (Sigma-Aldrich, USA). Results were mean values from three replicate analyses of the same sample. All samples and solutions were filtered through 0.45-μm nylon filters (Millipore, India) before analysis by HPLC. Plain mobile phase was used as a control for identification of blank peaks.

## Electronic supplementary material

Supplementary Information

## Data Availability

The RNA-seq raw reads of four samples have been submitted in NCBI’s Sequence Read Archive (PRJNA666394).
