# Author Correction: Transcriptome analysis provides insight into prickle development and its link to defense and secondary metabolism in *Solanum viarum* Dunal

**DOI:** 10.1038/s41598-021-95476-8

**Published:** 2021-08-03

**Authors:** Shatrujeet Pandey, Ridhi Goel, Archana Bhardwaj, Mehar H. Asif, Samir V. Sawant, Pratibha Misra

**Affiliations:** 1 Council of Scientific and Industrial Research - National Botanical Research Institute, Lucknow, 226001 India; 2 Academy of Scientific and Innovative Research (AcSIR), Ghaziabad, 201002 India

Correction to: *Scientific Reports* 10.1038/s41598-018-35304-8, published online 20 November 2018

The Data Availability section in the original version of this Article was omitted.

It now appears as below:

“The RNA-seq raw reads of four samples have been submitted in NCBI’s Sequence Read Archive (PRJNA666394).”

The original Article has been corrected.

