# Supplementary material for: Transcriptome analysis provides insight into prickle development and its link to defense and secondary metabolism in Solanum viarumDunal
Source: Sci Rep. 2018 Nov 20;8:17092. doi: 10.1038/s41598-018-35304-8 (PMC6244164; doi:10.1038/s41598-018-35304-8)
Supplement: Supplementary file 1 — Supplementary Information [file 41598_2018_35304_MOESM1_ESM.pdf]

Transcriptome analysis provides insight into prickle development and its link to defense and secondary metabolism in *Solanum viarum* Dunal

Shatrujeet Pandey[1,2], Ridhi Goel[1], Archana Bhardwaj[1], Mehar H. Asif[1], Samir V. Sawant[1,*], Pratibha Misra[1*]

[1]Council of Scientific and Industrial Research - National Botanical Research Institute, Lucknow-226001, India

[2]Academy of Scientific and Innovative Research (AcSIR), New Delhi-110 001, India

***Corresponding author** samirsawant@nbri.res.in; pratibhamisra@nbri.res.in;

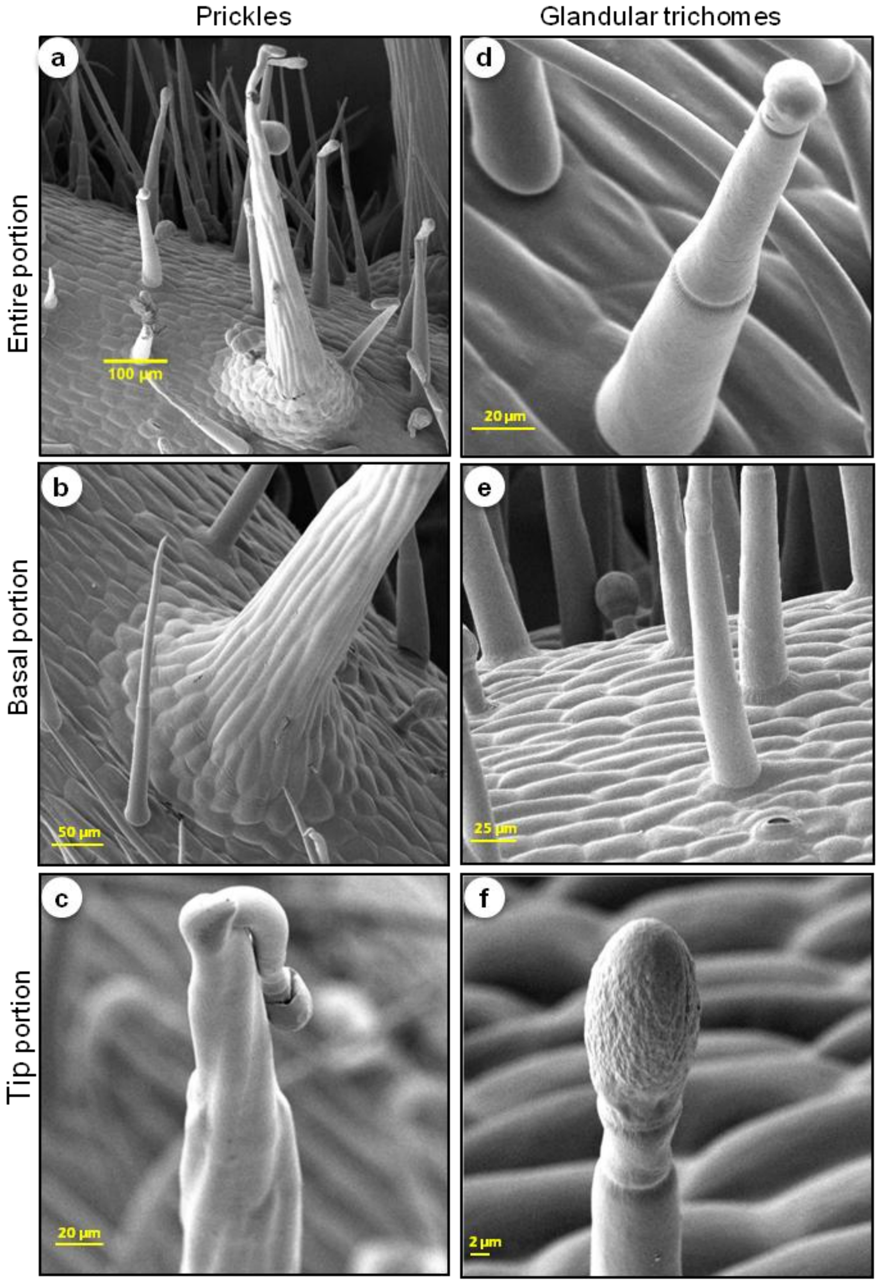

Supplementary Figure S1. Morphological similarity and differences between glandular prickle and glandular trichome of *S. viarum*. (a) Complete prickle. (b) Basal portion of prickle. (c) Tip portion of prickle showing GT attached to prickle. (d) Multicellular glandular trichome. (e) Base of trichomes. (f) Head of GTs.

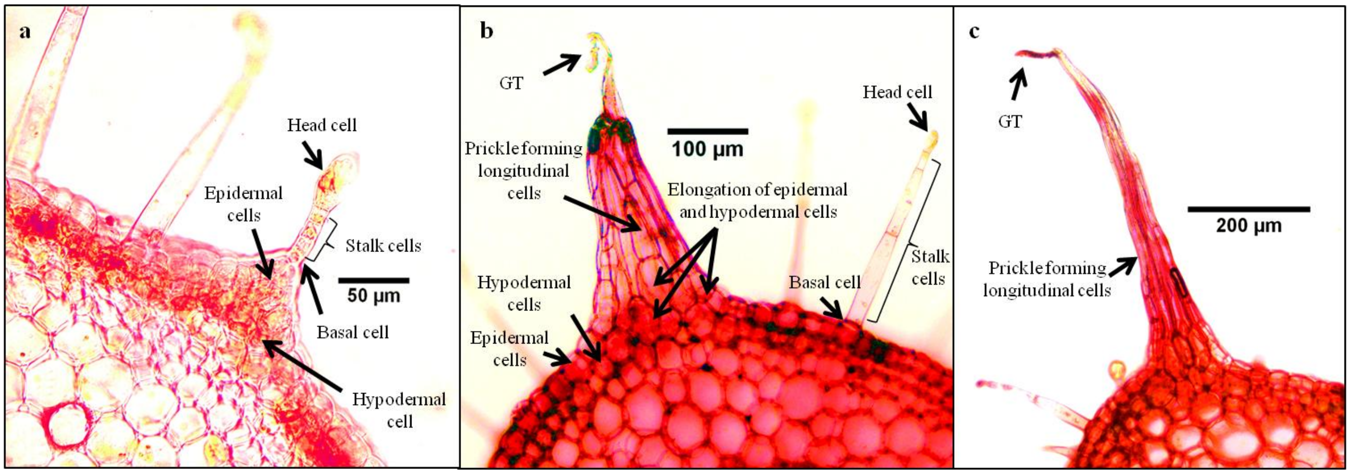

Supplementary Figure S2. T. S. of prickly stem showing different stages of prickle development in *S. viarum*. (a) Initiation of prickle formation beneath the basal cell of GT. (b) Developing prickle with GT attached to the tip. **(c)** Mature prickle with GT attached to the tip.

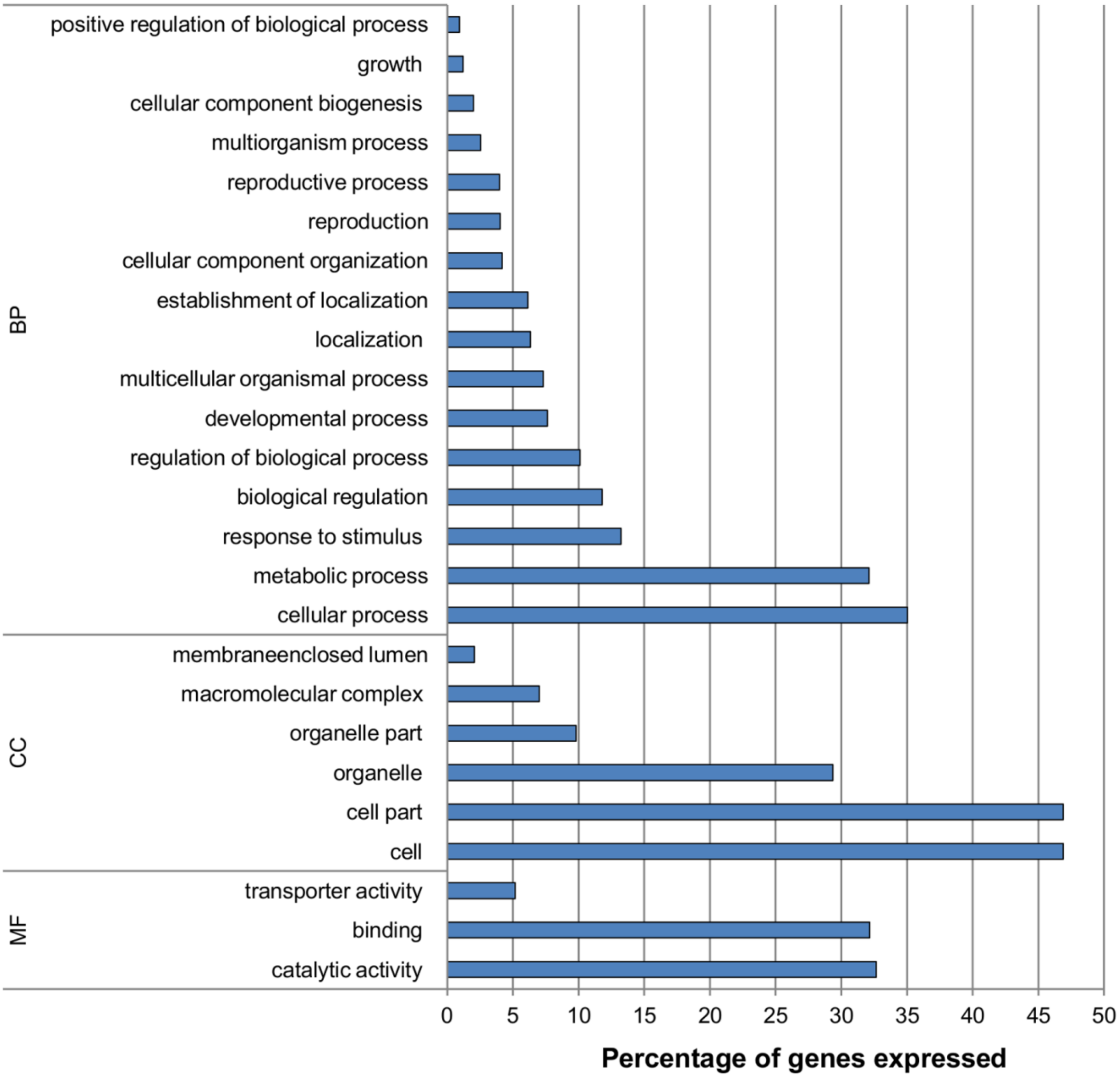

Supplementary Figure S3. Functional categorization of genes expressed in the epidermis of *S. viarum* through GO analysis.

a

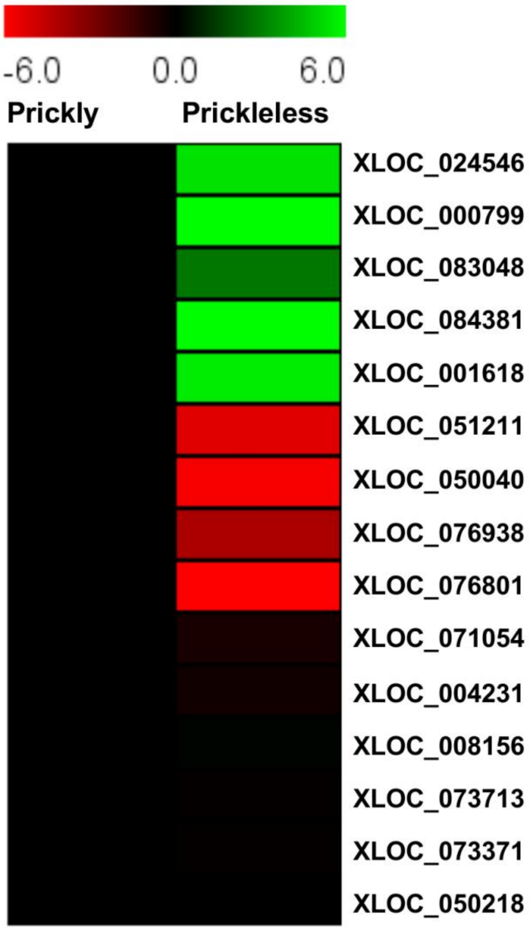

b

Gene_ids	FPKM	
	Prickly	Prickleless
XLOC_024546	4.73284	50.2076
XLOC_000799	0.580061	7.16596
XLOC_083048	0.254895	5.44907
XLOC_084381	0	103.257
XLOC_001618	0.928913	31.6138
XLOC_051211	13.9442	0.852679
XLOC_050040	3.06609	0.435983
XLOC_076938	6.31052	0
XLOC_076801	16.272	0
XLOC_071054	2.94642	0.120521
XLOC_004231	28.5467	28.9223
XLOC_008156	5.30996	5.29501
XLOC_073713	101.811	102.009
XLOC_073371	116.777	120.327
XLOC_050218	51.2437	51.4476

Supplementary Figure S4. qRT-PCR of some randomly selected highly up-regulated, down-regulated and similarly expressed genes for validation of transcriptome data of *S. viarum*. (a) Relative expression pattern analyzed through qRT-PCR. (b) FPKM based digital gene expression pattern analyzed through RNA-seq.

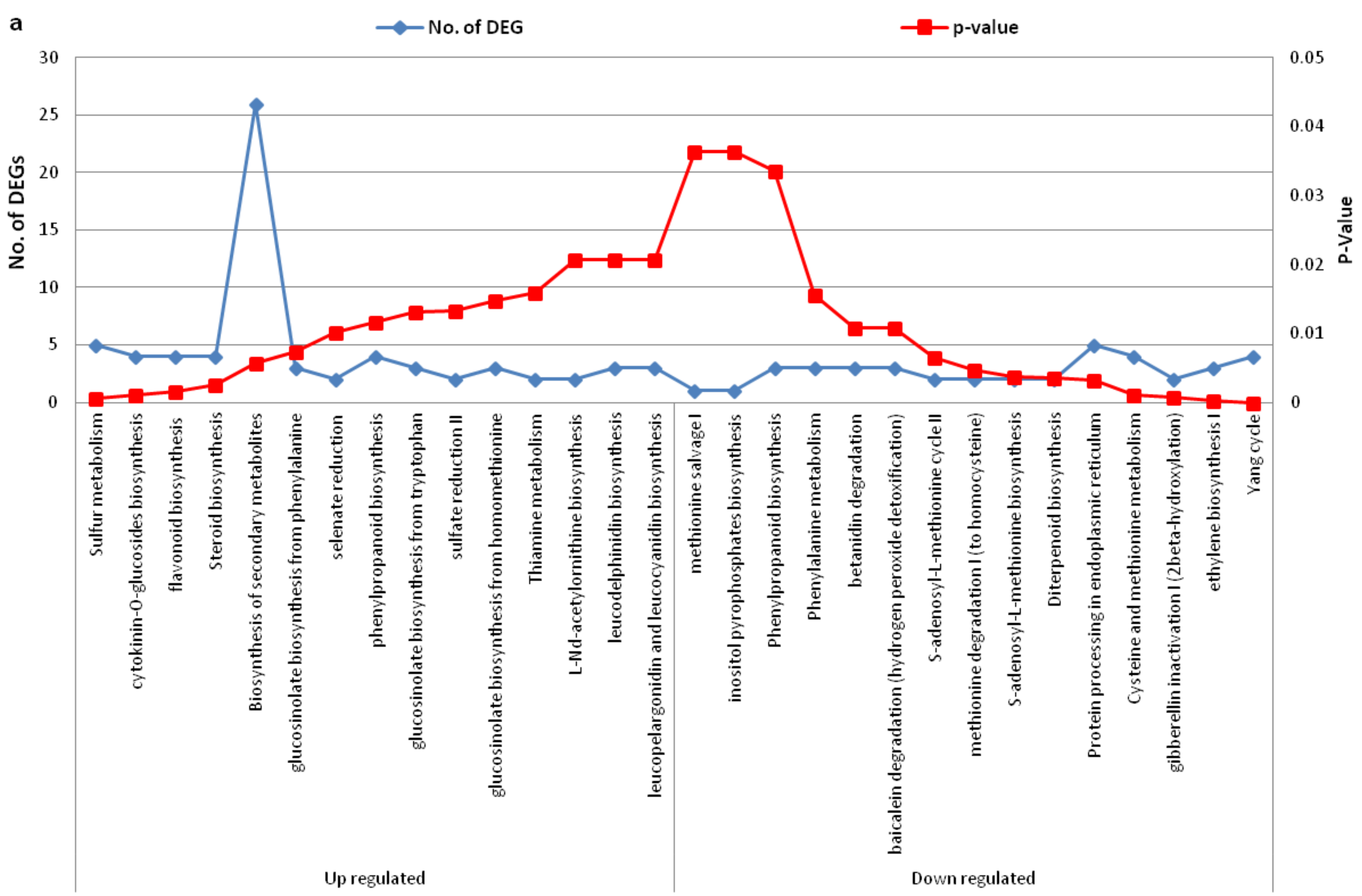

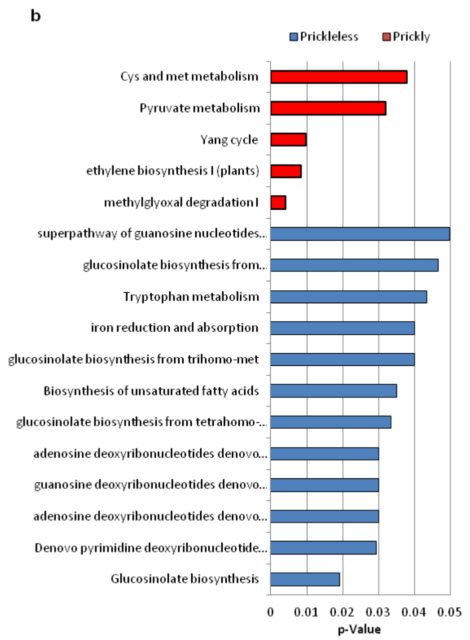

Supplementary Figure S5. Metabolic pathways altered in prickleless mutant of *S. viarum*. (a) Top 15 up and 15 down regulated pathways. (b) Uniquely expressed genes enriched to metabolic pathways significantly altered in prickly and prickleless mutant. Pathways with p-value ≤0.05 were considered statistically significant.

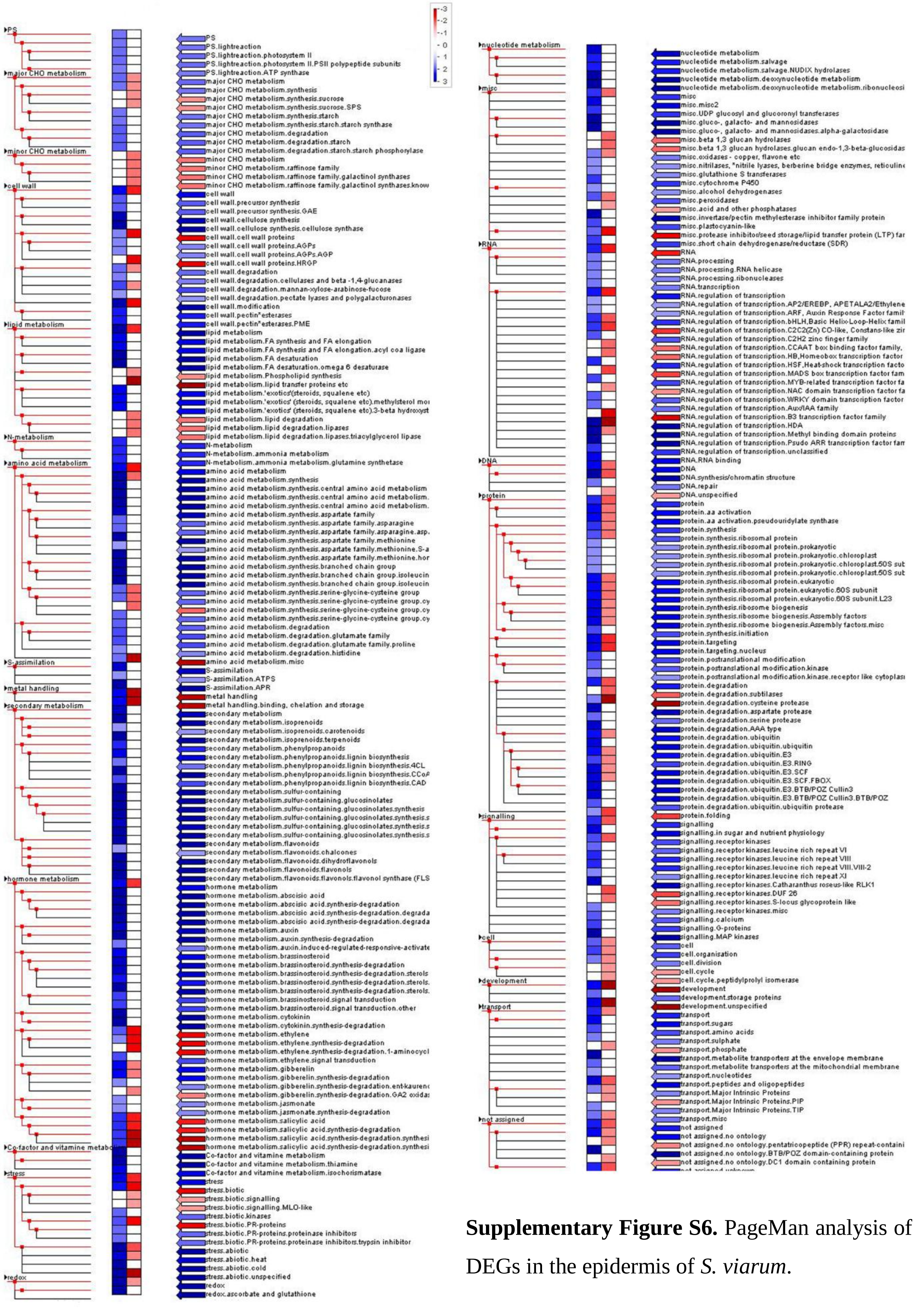

Supplementary Figure S6. PageMan analysis of DEGs in the epidermis of *S. viarum*.

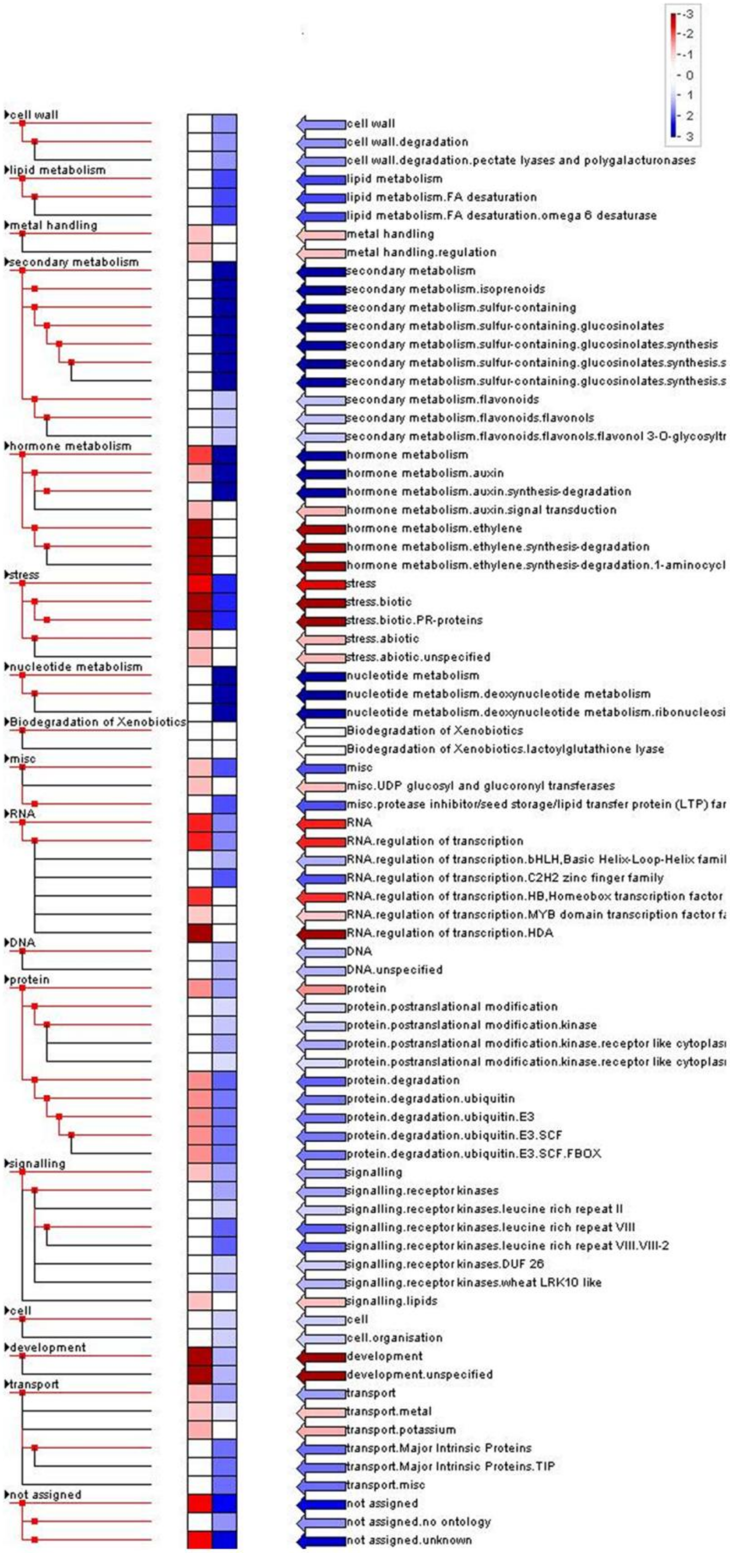

Supplementary Figure S7. PageMan analysis of uniquely expressed genes in epidermis of prickly or prickleless mutant of *S. viarum*.

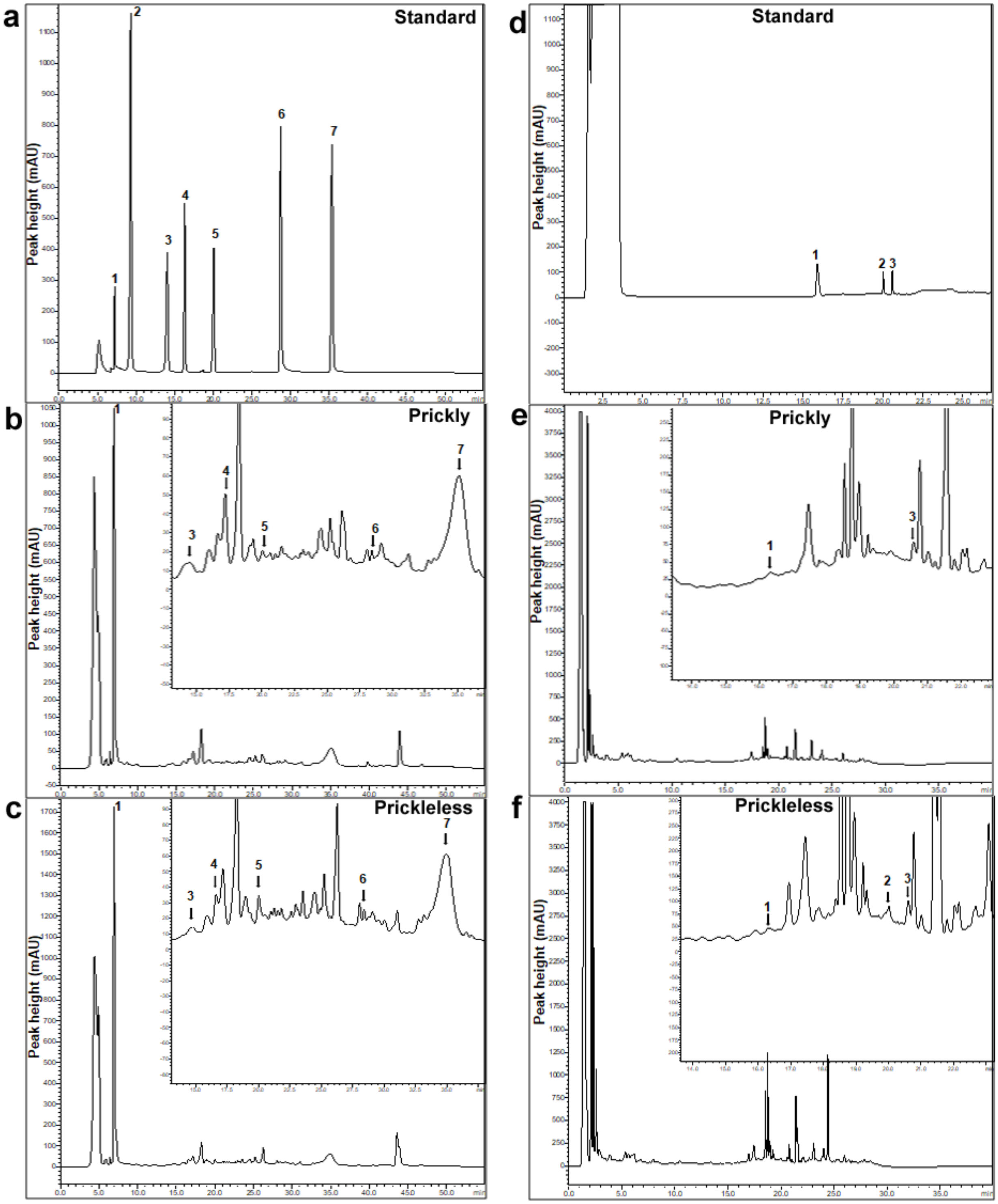

Supplementary Figure S8. HPLC chromatogram of the standard and crude extract of prickly and prickleless *S. viarum*. a-c) phenolics and flavonoid [gallic acid -1, chlorogenic acid -2, caffic acid-3, rutin-4, ferulic acid-4, quercitin-6, kaempferol-7. (d-f) alkaloid [α-solanine-1, solanidine-2, solasodine-3].

Supplementary Table S1. Summary of the transcriptome sequencing, *de novo* assembly and annotation of *S. viarum*.

	Prickly		Prickleless	
	**R1**	**R2**	**R1**	**R2**
**No. Of Reads**	18175222	15851932	17096127	13586099
**No. of HQ reads**	17444985 (95.98%)	15153863 (95.60%)	16429445 (96.10%)	12992487 (95.63%)
**No. of Contigs**	150880		142861	
**Largest Contig (bp)**	59569		46421	
**Smallest contig(bp)**	201		201	
**Average Length (bp)**	1126.75		1117.51	
**GC%**	46.11%		46.70%	
**NR Annotated transcript**	73189		70970	
**No. of trancripts after clustering**	156926			
**Largest Contig**	59569			
**Smallest contig**	201			
**Average Length**	1110.9			
**GC%**	39.30%			
**TAIR Annotated transcripts**	62975			
**TAIR Unannotated transcripts**	93951			
**Tomato Annotated transcripts**	67752			
**Tomato Unannotated transcripts**	89174			

Supplementary Table S4. List of the top 15 up and 15 down-regulated genes in epidermis of prickly vs. prickleless mutant of *S. viarum.*

Gene_Ids	Prickly vs. Prickleless (fold change)	p-value	Plant GD ID	Description	TAIR10 Ids	Tomato Ids
XLOC_064742	-5.118538307	0.00005	YCX91_PHAAO	Uncharacterized protein ORF91	NA	NA
XLOC_079911	-4.697050957	0.00005	NA	NA	NA	NA
XLOC_066158	-4.656741736	0.00005	YCF1A_MARPO	Putative membrane protein ycf1 N-terminal part; AltName: ORF 1068;	NA	NA
XLOC_071054	-4.611606642	0.00005	C5XHE6_SORBI	Putative uncharacterized protein Sb03g011550	NA	NA
XLOC_065300	-4.596655786	0.00005	Q40502_TOBAC	Extensin	NA	NA
XLOC_051211	-4.031518631	0.00005	B9HID6_POPTR	DEFORMED ROOTS AND LEAVES 1 family protein	AT1G13870.1	Solyc08g008250.1.1
XLOC_076801	-4.024319679	0.00005	F6HF70_VITVI	Putative uncharacterized protein	AT2G03440.1	Solyc04g005480.1.1
XLOC_067196	-3.927747592	0.00015	F6HH27_VITVI	Putative uncharacterized protein	AT3G22440.1	Solyc11g071340.1.1
XLOC_062280	-3.897727126	0.00035	Q0MX19_ARAHY	Leucine rich protein	NA	NA
XLOC_085802	-3.82662512	0.00005	E3W9P7_TOBAC	Putative PR-10 type pathogenesis-related protein	AT1G70890.1	Solyc04g007010.2.1
XLOC_065725	-3.796462781	0.00005	B3VI97_SOLDE	Putative disease resistance protein CC-NBS-LRR (Fragment)	AT3G14460.1	Solyc08g007630.1.1
XLOC_051029	-3.684004197	0.0001	Q93YD2_TOBAC	Putative translocation transactivator	NA	Solyc08g044450.1.1
XLOC_042610	-3.682356673	0.00005	Q39467_CAPAN	Sn-2 protein (MLP-like protein 34)	AT5G28010.1	Solyc04g007770.2.1
XLOC_024732	-3.652715931	0.00005	D7LTE8_ARALL	Amino acid transporter, putative	NA	NA
XLOC_038231	-3.596861021	0.00005	E7D4U5_TOBAC	Cysteine protease	AT5G45890.1	Solyc02g076910.2.1
XLOC_084381	6.690095778	0.00005	F2CZR9_HORVD	Predicted protein (Uncharacterized protein)	NA	NA
XLOC_007885	6.004369091	0.00005	B9GNI9_POPTR	Uncharacterized protein	AT5G48850.1	Solyc06g007970.2.1
XLOC_031857	5.987186837	0.00005	B9S545_RICCO	Putative uncharacterized protein	AT5G48850.1	Solyc06g007970.2.1
XLOC_002160	5.647052243	0.00095	B3SP99_CAPAN	Fatty acid desaturase	AT3G12120.2	Solyc01g006430.2.1
XLOC_085535	5.481334466	0.00005	PRS2_SOLTU	Pathogenesis-related protein STH-2	NA	Solyc09g091000.2.1

XLOC_023238	5.201494901	0.00005	A5BSN3_VITVI	Putative uncharacterized protein	AT1G22400.1	Solyc04g074330.2.1
XLOC_005604	5.10395771	0.00005	Q9LM08_CAPAN	Pepper esterase	AT5G62180.1	Solyc06g035520.2.1
XLOC_001618	5.088867165	0.00005	B9HUS6_POPTR	Homocysteine S-methyltransferase 3 family protein	AT3G63250.1	Solyc09g082460.2.1
XLOC_034413	5.07700713	0.0002	B9RP37_RICCO	Putative uncharacterized protein	NA	NA
XLOC_018784	4.961102747	0.0003	A2Q1J3_MEDTR	DUF506 family protein	AT1G12030.1	Solyc11g012120.1.1
XLOC_015461	4.911130691	0.00005	D7MJF9_ARALL	Putative uncharacterized protein	NA	NA
XLOC_030037	4.709704193	0.00005	D5KXD2_SOLLC	Beta caryophyllene/humulene synthase (Caryophyllene/alpha-humulene synthase)	AT3G14540.1	Solyc06g059930.2.1
XLOC_019256	4.564999303	0.00005	C6TGT6_SOYBN	Uncharacterized protein	AT3G63030.1	Solyc06g084400.2.1
XLOC_022696	4.457186326	0.00005	B9TIC7_RICCO	Cytochrome P450, putative (EC 1.14.13.76) (Fragment)	NA	NA
XLOC_083048	4.418035045	0.0002	P93789_SOLTU	UDP-galactose:solanidine galactosyltransferase	AT2G36790.1	Solyc07g043490.1.1

Supplementary Table S6. List of the primers used for qRT-PCR analysis.

Selected primers used for transcriptome data validation				
		Primer Sequence 5' to 3'		
**Sl. No.**	**Gene_Ids**		**Forward**	**Reverse**
1	XLOC_024546		CCATTCCACCAGCCCATCT	CGCCCACATCGTTGTTTG
2	XLOC_000799		CAAAAAATGAGCACGTGAGAAAA	CGCCCCCAAGCTAACTTCTT
3	XLOC_083048		CCAGCCTACCATTGATGACGA	TGGCAGTGTTGAAGCTCTCA
4	XLOC_084381		GGAAAGCCGGTGACTCTCAA	TCGTCTGCTCTGCTTCGTTC
5	XLOC_001618		AGGCAACTCTTCAGGGATTT	TCTCTACGCTTCTTTTGAGCA
6	XLOC_051211		TGTTTGGCTAGAGCAGCAGG	ACGGTTCTCCTCTCTCACGA
7	XLOC_050040		GCTTCATTGCGTGCTTGTTG	TATCGCCTTATCTTCACCATTCC
8	XLOC_076938		CGAAAGGAGATAGAGAAGATGGAAGA	CCTTTTCTAACTCCACCGTGTGT
9	XLOC_076801		CGGTTGTTGTGGTGGTAGA	ATTCGTCGGAAAAGCAGAG
10	XLOC_071054		TCAGGCATTGAGTTTGTTGA	CTCGGGCATTGTTGTTTTAC
11	XLOC_004231		TGCGAAGCAGAGACCATACAGT	GGAGGACCCAGAACGGAATT
12	XLOC_008156		GGTGATTTCTTGGTCTCCTTTCC	CACGCTAAGGAGGCTGCAA
13	XLOC_073713		GCATTCAACCCATCAGAGCAA	TTGTCTTCCCACCACTCTGTGA
14	XLOC_073371		CGGCTGTGGTGGTGAAAGAG	TCTGGCAGGGCGTGATCT
15	XLOC_050218		CCACAGCGGGCAACAGTAA	TCTCCGTGGTGGTTTTTAGGTT
**Selected primers used for the expression analysis of transcription factors**				
**Sl. No.**	**Gene_Ids**	**Gene names**	**Forward**	**Reverse**
1	XLOC_067399	REM	TTGAAGCTGAATGGACATCG	CCCAATTGCACATTATGATCC
2	XLOC_040694	MSM1	TGGACTCATTGTTTTTTCTGCTACTG	CCCTTTCTGACTGCGTCTTGT
3	XLOC_050040	C2C2-CO-like	GCTTCATTGCGTGCTTGTTG	TATCGCCTTATCTTCACCATTCC
4	XLOC_051211	DRL1	TGTTTGGCTAGAGCAGCAGG	ACGGTTCTCCTCTCTCACGA
5	XLOC_027183	Homeobox	TCTTCCATCTAGCTCGCCTG	TGCTAAAGAGTTGGGTGTTCC
6	XLOC_075558	R2R3-MYB	ATGTGGTCATATTCCTCTGGTTG	GGACCTCTCTTCAATCCAACTCT
7	XLOC_035637	HAP2	GACGAATGGAAGCATCAACGA	TGCATACATCAGACTGCACAAC
**Selected primers used for expression analysis of secondary metabolite pathways**				
1	XLOC_009213	FLS1	ATTTGTGAGCCCCTTCCAC	TTGCACCCATTTTTCTCCA
2	XLOC_034589	CHS	GAAGGTCGAAGCCCAAGAA	GCCAAGCCAAGTCCAAAAT
3	XLOC_084597	CFI	TTCCATGTCCCATCAAAGGT	TGAAGTGGTGGTGGATGAAA
4	XLOC_025921	COMT	CAAAAAGCGTGGTTGGAGTT	TTTTGGTTCTTCCACGCAAT
5	XLOC_052049	4CL	GGCAAGGTCTCTCCACTTTCAG	TGAGGAAACAGACCGGTATGG
